# Inkjet printed circuits based on ambipolar and p-type carbon nanotube thin-film transistors

**DOI:** 10.1038/srep39627

**Published:** 2017-02-01

**Authors:** Bongjun Kim, Michael L. Geier, Mark C. Hersam, Ananth Dodabalapur

**Affiliations:** 1 Microelectronics Research Center, The University of Texas at Austin, Austin, Texas 78758, United States; 2 Department of Materials Science & Engineering and Department of Chemistry, Northwestern University, Evanston, Illinois 60208, United States

## Abstract

Ambipolar and p-type single-walled carbon nanotube (SWCNT) thin-film transistors (TFTs) are reliably integrated into various complementary-like circuits on the same substrate by inkjet printing. We describe the fabrication and characteristics of inverters, ring oscillators, and NAND gates based on complementary-like circuits fabricated with such TFTs as building blocks. We also show that complementary-like circuits have potential use as chemical sensors in ambient conditions since changes to the TFT characteristics of the p-channel TFTs in the circuit alter the overall operating characteristics of the circuit. The use of circuits rather than individual devices as sensors integrates sensing and signal processing functions, thereby simplifying overall system design.

Inkjet printing technology has emerged as one of the most promising device fabrication methods for low-cost electronics^1^. Its direct, additive patterning capability significantly reduces material waste and processing time compared to conventional subtractive patterning methods such as photolithography and etching/lift-off. In addition, patterns to be printed can be easily altered without replacing physical masks such as costly photo or shadow masks since patterns are digitally designed. Various functional materials, including polymers^2^, amorphous oxides^3^,^4^,^5^,^6^, single-walled carbon nanotubes (SWCNTs)^5^,^6^,^7^,^8^, and emerging low-dimensional materials^9^,^10^,^11^,^12^, have been deposited by inkjet printing and have shown great promise in electronic applications. Recently, it also has been shown that a printing technique can be employed to produce aligned graphene nanoribbons in large area^13^. In particular, semiconducting SWCNTs have been extensively explored in diverse disciplines due to their superb electrical, mechanical, and chemical properties^14^,^15^,^16^. Such properties have been highlighted in high performance transistors and circuits^5^,^17^,^18^, flexible electronics^15^,^16^, and chemical/biosensors^19^,^20^,^21^,^22^.

Semiconducting SWCNTs are intrinsically ambipolar materials; however, they typically exhibit unipolar p-type behavior under ambient conditions because of adsorbed oxygen and moisture^16^,^23^,^24^. In order to construct complementary circuits, which are the most robust and power efficient circuit technology for integrated circuits, materials with different doping types are needed for semiconductors in thin-film transistor (TFT) based circuits. One method for achieving this goal is to combine n-channel semiconducting oxides with p-channel SWCNTs^5^,^6^,^25^. Alternatively, SWCNTs can realize n-type or ambipolar behavior by employing approaches such as chemical doping^26^,^27^,^28^,^29^,^30^, covering with appropriate dielectrics^31^,^32^,^33^,^34^,^35^,^36^,^37^,^38^,^39^, and using low work function metal contacts^40^,^41^,^42^. Several basic complementary or complementary-like circuits have been successfully demonstrated by employing the same semiconducting channel materials, SWCNTs, for different conductivity type of transistors^8^,^26^,^27^,^29^,^32^,^33^,^34^,^37^,^38^,^39^,^43^. However, none of these circuits have been fabricated by photo or shadow mask-free methods. It should also be noted that complementary SWCNT circuit sensors have not yet been achieved, while numerous SWCNT transistor based sensors have been reported previously^19^,^20^,^21^,^22^. Complementary ring oscillator (ROSC) circuit based sensors possess advantages over single transistor based sensors such as a natural recovery of the drain current in TFTs^44^. The decrease in drain current over time in TFT-based sensors can make it difficult to extract the changes due to chemical responses. This can be mitigated in ROSC based sensors by naturally cycling each transistor gate bias, between *V*_*DD*_ and GND. ROSC based sensors can also provide already digitized data that can be measured by the frequency variations.

Very recently, a few complementary-like circuits with ambipolar and p-type SWCNT TFTs have been demonstrated^8^,^38^,^43^. In this paper, we present inkjet printed complementary-like circuits based on ambipolar and p-type SWCNT TFTs fabricated by photo/shadow mask-free methods. We also show that these circuits are sensitive to chemical vapors, suggesting their use in the design of chemical sensors. Inkjet printing is employed in a more complex circuit fabrication scheme, rather than unit TFTs, where all seven layers are deposited or patterned by inkjet printing. In these circuits, ambipolar and p-type TFTs in the circuits are implemented on different planes as a three-dimensional integrated circuit demonstration, which is not possible with conventional semiconductor materials. A shared Al_2_O_3_ layer is utilized for conductivity type conversion (from p- to ambipolar-type) and gate dielectric layers for p-TFTs whose channels are open to the air. Various complementary-like circuits, which include inverters, ROSCs, and NAND gates, are demonstrated. In addition, SWCNTs are exposed to acetone vapor, which is an exemplary polar analyte, to investigate the effects of chemical vapors on these circuits.

## Results and Discussion

**Figure 1:**
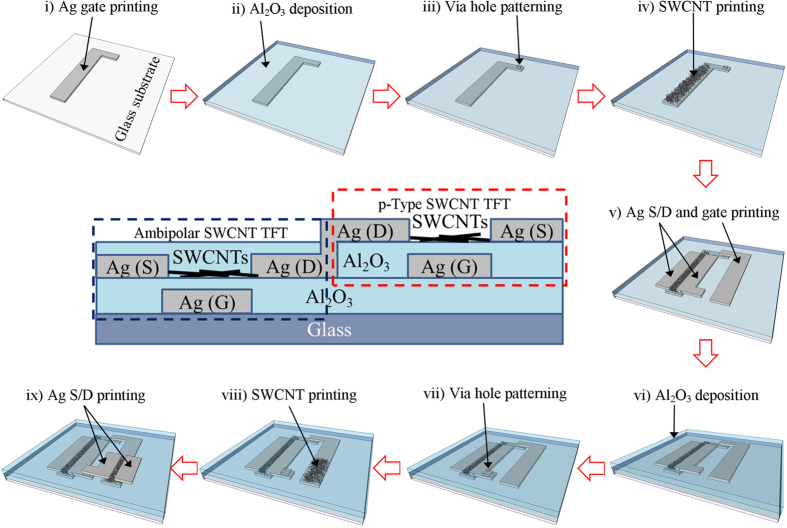
Process flow of an inkjet printed inverter based on ambipolar and p-type SWCNTs, where ambipolar and p-type TFTs are on different planes. (i) Ag gate printing for the ambipolar TFT. (ii) Al_2_O_3_ gate dielectric deposition by ALD for the ambipolar TFT. (iii) Acetone printing on the photoresist coated sample for via hole patterning, followed by wet etching and photoresist removal. (iv) SWCNT printing for the ambipolar semiconducting channel. (v) Ag source/drain (for the ambipolar TFT) and gate (for the p-TFT) printing. (vi) Al_2_O_3_ gate dielectric deposition by ALD for the p-TFT. (vii) Acetone printing on the photoresist coated sample for another via hole patterning, followed by wet etching and photoresist removal. (viii) SWCNT printing for the p-type semiconducting channel. (ix) Ag source/drain printing for the p-TFT. The middle panel shows a schematic cross-section of the completed inkjet printed inverter.

Figure 1 illustrates the fabrication process of an inkjet printed inverter based on ambipolar and p-type SWCNTs and its schematic cross-section. Ambipolar and p-type SWCNT TFTs were integrated on the same glass substrate using inkjet printing. The second Al_2_O_3_ layer was deposited to convert as-printed p-type SWCNTs to ambipolar SWCNTs^32^,^36^ and to serve as gate dielectrics for p-type SWCNT TFTs whose semiconducting channels were open to the air. The completed devices were composed of seven layers, which were either deposited as uniform coatings or patterned by inkjet printing without the use of shadow masks or photolithography.

**Figure 2:**
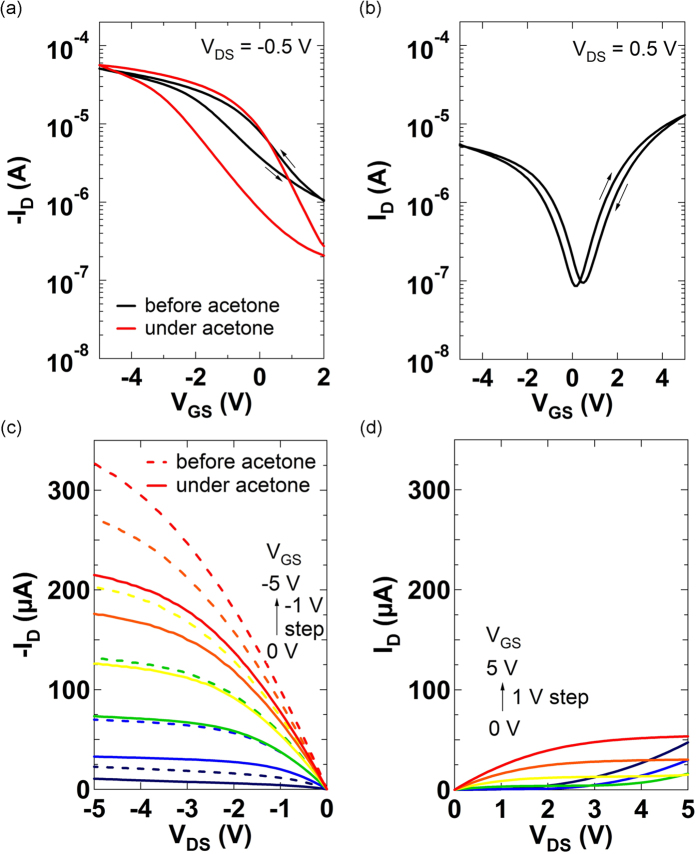
(**a**) Transfer characteristics (*I*_*D*_ − *V*_*GS*_) of the p-type SWCNT TFT at *V*_*DS*_ = −0.5 V before and under acetone vapor exposure (*L* = 65 μm, *W* = 1406 μm). (**b**) Transfer characteristics of the ambipolar SWCNT TFT at *V*_*DS*_ = 0.5 V (*L* = 37 μm, *W* = 719 μm). (**c**) Output characteristics (*I*_*D*_ − *V*_*DS*_) of the p-type SWCNT TFT before and under acetone vapor exposure. (**d**) Output characteristics of the ambipolar SWCNT TFT in n-channel operation mode.

Figure 2a and b show transfer characteristics (*I*_*D*_ − *V*_*GS*_) of the p-type (before and under acetone vapor exposure) and ambipolar SWCNT TFTs, respectively. Device characteristic parameters (hole and electron mobilities for p- and ambipolar TFTs, respectively, log (*I*_*on*_/*I*_*off*_), threshold voltage (*V*_*th*_), subthreshold swing (S. S.)) of the ambipolar and p-type SWCNT TFTs in forward sweeps are listed in Table 1. The parameters for the ambipolar TFTs were extracted at n-channel operation mode (at positive *V*_*GS*_ and *V*_*DS*_) since the ambipolar TFTs were employed as pull-down TFTs in our complementary-like circuits. The mobility values were calculated using equation (1):


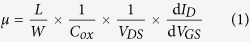


where *L* is the channel length, *W* is the channel width, and *C*_*ox*_ = 161 nF cm^−2^ is the areal capacitance of the dielectrics. Metal (Ti/Au)-insulator (Al_2_O_3_)-metal (Ni) capacitors were fabricated to measure *C*_*ox*_ as described in previous work^6^.

SWCNT TFTs whose channels are exposed to air typically show p-type behavior with hysteresis (Fig. 2a) due to adsorption of oxygen and moisture^16^,^23^,^24^. However, ambipolar SWCNT TFTs, whose channels are covered with Al_2_O_3_, exhibit ambipolar behavior^32^,^36^ as shown in Fig. 2b, where both electrons and holes are transported in a single device and the magnitudes of the electron and hole currents are determined by bias conditions. In order to investigate the effects of polar molecule vapors, acetone was delivered to the channel of the p-TFT. Under acetone vapor exposure, the mobility value increased and *I*_*off*_ and S. S. decreased compared to corresponding values before acetone vapor exposure. These favorable changes in device characteristics are attributed to the polar molecule’s partial neutralization of charged impurities/defects in the channel^45^,^46^. On the other hand, the hysteresis window in Fig. 2a becomes larger under acetone vapor exposure due to the acetone molecule reorientation in response to the electric field direction^46^. Such noticeable device characteristic changes under acetone vapor exposure were not observed in the ambipolar SWCNT TFTs since the channels of the ambipolar TFTs were encapsulated by Al_2_O_3_. It also has been shown that no observable changes occur when a non-polar molecule, such as hexane, is delivered to the channel of p-type SWCNT TFTs^46^. Figure 2c and d show output characteristics (*I*_*D*_ − *V*_*DS*_) of the p-type (before and under acetone vapor exposure) and ambipolar SWCNT TFTs, respectively.

**Figure 3:**
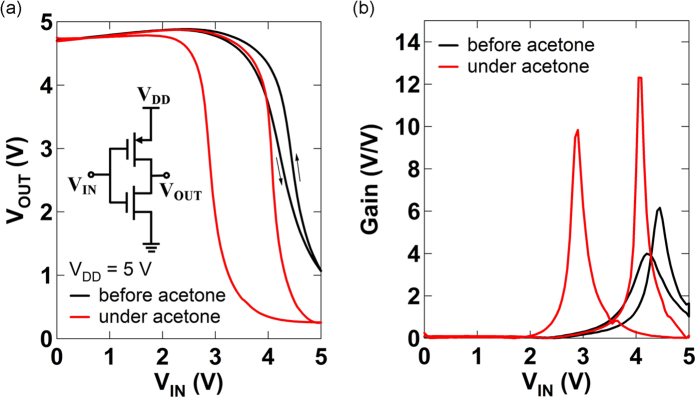
(**a**) Voltage transfer characteristics of the inverter based on ambipolar and p-type SWCNT TFTs before and under acetone vapor exposure. Inset shows a circuit diagram of the inverter. (**b**) DC gain (|d*V*_*OUT*_/d*V*_*IN*_|) of the inverter before and under acetone vapor exposure.

The p-type TFT (as a pull-up transistor) and the ambipolar TFT (as a pull-down transistor) were connected to construct a complementary-like inverter as shown in inset of Fig. 3a. Voltage transfer characteristics (VTCs) (Fig. 3a) of the inverter based on p-type and ambipolar SWCNT TFTs show non-constant *V*_*OUT*_ (slightly increasing with increase of *V*_*IN*_) at low *V*_*IN*_, while almost constant *V*_*OUT*_ is shown at high *V*_*IN*_. This is because the ambipolar TFT shows super-linear current increase (due to minority carrier injection) instead of complete turn-off while the p-TFT operates in the linear regime at low *V*_*IN*_, whereas the p-TFT shows almost constant off-current while the ambipolar TFT operates in the linear regime at high *V*_*IN*_ as shown in Fig. 2c and d. VTCs of inverters composed of two ambipolar TFTs show different curve shapes where *V*_*OUT*_ at both low and high *V*_*IN*_ slightly increase with increase of *V*_*IN*_^17^,^35^,^36^,^47^. The static power dissipation in the inverter composed of p-type and ambipolar TFTs is lower than that in the inverter composed of two ambipolar TFTs. The power dissipation decreases at high *V*_*IN*_, where p-TFT is turned off, in the inverter based on p-type and ambipolar TFTs (Supplementary Fig. S1), whereas the power dissipation increases at both low and high *V*_*IN*_ in the inverter based on only ambipolar TFTs^5^.

Effects of the polar acetone vapor on the inverter circuit were also investigated by delivering acetone vapor to the channel of the p-TFT. VTCs under acetone vapor exposure show lower *V*_*OUT*_ (closer to GND) at high *V*_*IN*_ and shift to the left along the axis of *V*_*IN*_ with a larger hysteresis as shown in Fig. 3a. In the case of an ideal complementary inverter, VTCs show rail-to-rail swing (from *V*_*DD*_ to GND) without voltage loss at low and high *V*_*IN*_ since one transistor is turned off while the other transistor is turned on. In our case, the ambipolar characteristics in the pull-down transistor described above causes voltage loss at low *V*_*IN*_, and relatively high off-current in the p-TFT results in voltage loss at high *V*_*IN*_ before acetone vapor exposure. By lowering off-current in the p-TFT under acetone vapor exposure as shown in Fig. 2c, *V*_*OUT*_ (~0.3 V) at high *V*_*IN*_ is lowered compared to *V*_*OUT*_ (~1.1 V) at high *V*_*IN*_ before acetone vapor exposure. The shift of the VTC under acetone vapor exposure occurs because the magnitude of *I*_*D*_ in the p-TFT decreases upon acetone exposure as shown in Fig. 2c, while *I*_*D*_ in the ambipolar TFT remains the same. The larger hysteresis in VTCs under acetone vapor exposure is due to the hysteresis in the p-TFT under acetone vapor exposure as shown in Fig. 2a. These changes in the inverter characteristics are reversible if the device is left under ambient conditions for a few min without acetone vapor exposure (Supplementary Fig. S2). DC gains (|d*V*_*OUT*_/d*V*_*IN*_|) of the inverter are shown in Fig. 3b. The maximum DC gain under acetone vapor exposure shows more than two-fold improvement compared to the maximum DC gain before acetone vapor exposure because of the improved p-TFT characteristics under acetone vapor exposure. The power dissipation in the inverter decreases at high *V*_*IN*_ under acetone vapor exposure (Supplementary Fig. S1) due to the off-current decrease in the p-TFT under acetone vapor exposure.

**Figure 4:**
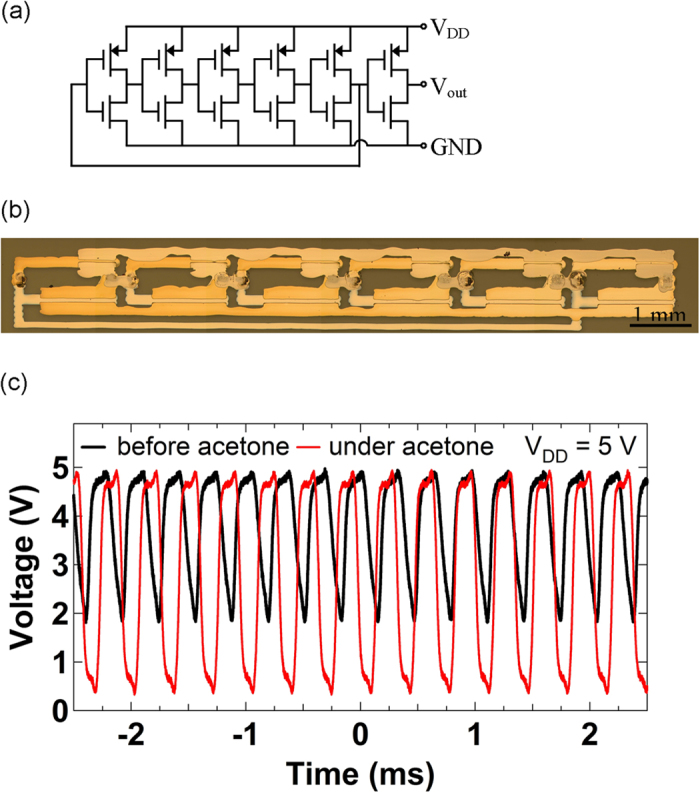
(**a**) Circuit diagram of a five-stage ROSC based on ambipolar and p-type SWCNT TFTs with a buffer stage. (**b**) Micrograph of the inkjet printed ROSC based on ambipolar (*L* = 59 μm, *W* = 1198 μm) and p-type (*L* = 32 μm, *W* = 609 μm) SWCNT TFTs. (**c**) Output signals of the ROSC before and under acetone vapor exposure.

Five-stage ROSCs, where five ambipolar and p-type SWCNT TFTs-based inverters are connected in a loop as shown in Fig. 4a, were fabricated by inkjet printing. Figure 4b shows a micrograph of the printed five-stage ROSC on a glass substrate. Figure 4c shows output signals of the ROSC at *V*_*DD*_ = 5 V before and under acetone vapor exposure. The tip of the syringe for acetone vapor delivery was positioned in close proximity to the channel of the p-TFT in the last stage of the ROSC. The ROSC operated at an oscillation frequency of 3.14 kHz with peak-to-peak voltage (V_pp_) of 3.16 V before acetone vapor exposure, but was changed to an oscillation frequency of 2.91 kHz with V_pp_ of 4.62 V under acetone vapor exposure as shown in Fig. 4c. Oscillation frequencies of the printed ROSC were mostly limited by long *L* and large overlaps between gate and source/drain (S/D) electrodes. The relatively large values of *L* and gate overlaps, in this work, were chosen to avoid device failures which may be caused by utilization of inkjet printing for electrodes patterning, thus the oscillation frequencies can be further increased by optimized design of the device^5^. The decrease in off-current of the p-TFT also enhances the swing of output signals of the ROSC as described in the case of the inverter under acetone vapor exposure. The enhanced voltage swing of the ring oscillator under exposure to acetone is largely a result of a favorable shift in threshold voltage of the p-TFT, which improves inverter performance (both voltage swing and noise margin). The use of circuits such as ring oscillators rather than discrete devices as sensors imparts concurrent signal processing functionality. In particular, the sensor response includes a frequency shift or a voltage amplitude change instead of only a current change in traditional discrete transistor sensors.

**Figure 5:**
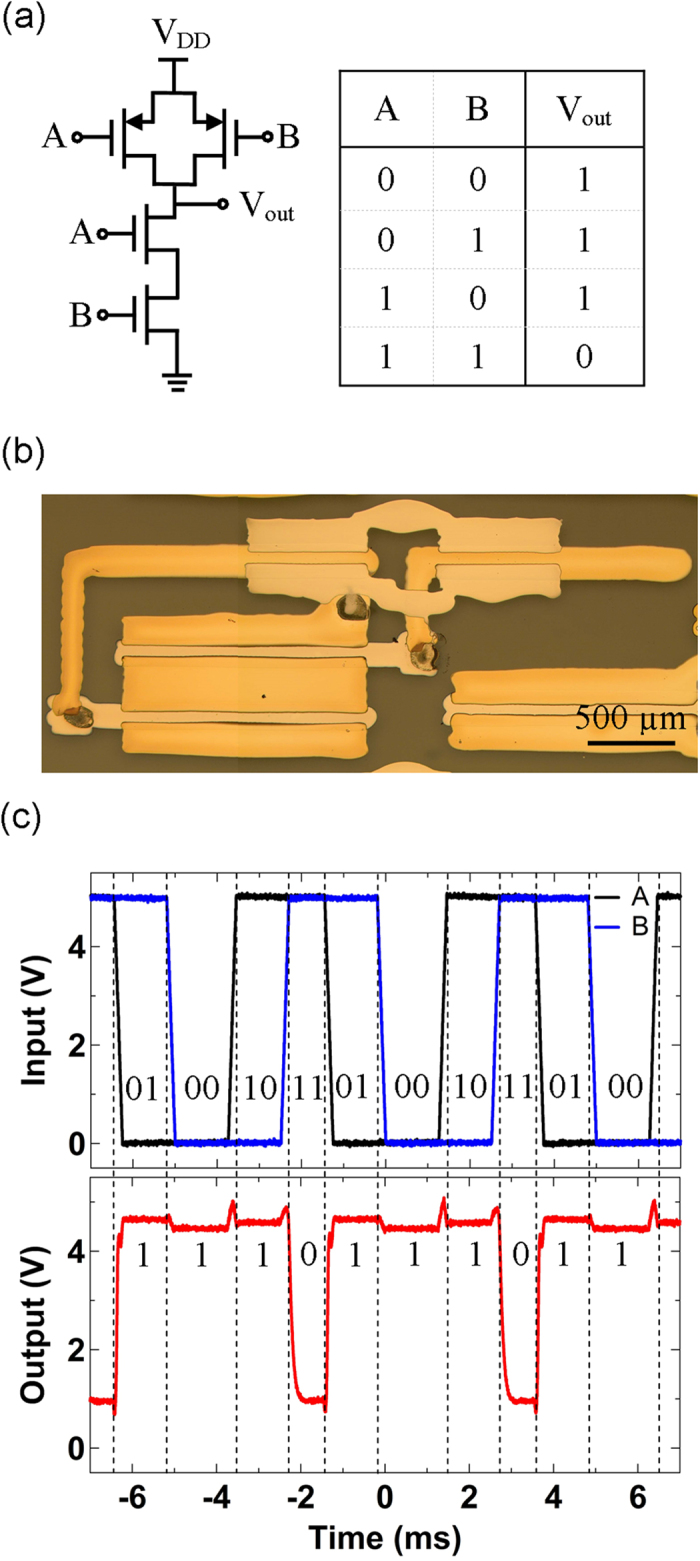
(**a**) Circuit diagram and truth table of a NAND gate based on ambipolar and p-type SWCNT TFTs. (**b**) Micrograph of the inkjet printed NAND gate based on ambipolar (L = 59 μm, W = 1400 μm) and p-type (L = 69 μm, W = 696 μm) SWCNT TFTs. (**c**) Dynamic response of the NAND gate when 200 Hz input signals A and B are applied.

NAND logic gates, which are composed of four printed TFTs (two p-TFTs connected in parallel and two ambipolar TFTs connected in series) as shown in the circuit configuration in Fig. 5a, were also fabricated to further demonstrate the potential of the combination of printed ambipolar and p-type SWCNT TFTs in logic circuits. Figure 5b displays an optical micrograph of the printed NAND logic gate on a glass substrate. Output signals of the NAND gate exhibit logic “0” only when both input signals are at logic “1”, and show logic “1” in all other input cases as demonstrated in Fig. 5c.

## Conclusion

Various complementary-like circuits have been realized by integrating ambipolar and p-type SWCNT TFTs three-dimensionally on the same glass substrate. Different doping conditions are achieved by employing the same channel material (SWCNTs) with an Al_2_O_3_ layer for ambipolar TFTs or exposure to air for p-TFTs. All patterns, which include electrodes, via holes, and semiconductors, in the circuits have been formed by cost-effective inkjet printing. In addition, the behavior of complementary-like ROSCs as chemical sensors has been investigated. Exposure to polar vapors such as acetone significantly alters the operating characteristics, which include changes in the voltage swing amplitude and oscillation frequency. The work presented here demonstrates that random network SWCNT semiconductors possess high potential for use in printed complementary/complementary-like circuits as well in chemically sensitive circuits.

## Methods

### Device Fabrication

Glass substrates (AF 32 eco, SCHOTT) were cleaned by sequential sonication in acetone, methanol, and 2-propanol for 3 min each. Commercial Ag nano-particle ink (SOLSYS EMD5603, Sun Chemical Corporation) was deposited by inkjet printing for bottom gate electrodes of ambipolar SWCNT TFTs after UV O_3_ surface treatment for 5 min on the substrates. After the gate printing, the substrates were placed on a hot plate in air at 200 °C for 30 min. An Al_2_O_3_ gate dielectric (43 nm) for ambipolar SWCNT TFTs was deposited by atomic layer deposition (ALD) (Cambridge Nanotech) at 220 °C. Photoresist (AZ 5209-E) serving as an etch mask was spin coated on the Al_2_O_3_ layer, then acetone was inkjet printed five times sequentially for via hole pattering. Via holes were created for connections between ambipolar and p-type TFT gate electrodes by wet etching with diluted buffered oxide etch (BOE) (H_2_O:BOE = 40:1) for 2 min. After removal of the remaining photoresist by acetone, the surface of the dielectric layer was treated with UV O_3_ for 10 min to promote wetting of SWCNT inks^48^. SWCNTs (with >98% semiconducting SWCNTs prepared by density gradient ultracentrifugation)^49^,^50^ dispersed in 1-cyclohexyl-2-pyrrolidone^51^ (Sigma-Aldrich) were inkjet printed on the dielectric layer for ambipolar semiconducting channels, followed by annealing on a hot plate in air at 200 °C for 30 min to remove residual solvent. Ag ink was inkjet printed to form S/D electrodes for ambipolar SWCNT TFTs, bottom gate electrodes for p-type SWCNT TFTs, and interconnects, followed by annealing on a hot plate in air at 200 °C for 30 min. A second Al_2_O_3_ layer (43 nm) was deposited on the samples by ALD at 220 °C for semiconductor conductivity conversion^36^ and p-type SWCNT TFT gate dielectrics. Another via hole patterning, for connections between ambipolar and p-type TFT drain electrodes as well as connections between discrete inverter input and output terminals in ROSCs, was performed by the same process described above. After UV O_3_ surface treatment for 10 min, SWCNTs were deposited by inkjet printing for p-type semiconducting channels, followed by annealing on a hot plate in air at 200 °C for 30 min. Finally, Ag ink was printed to form S/D electrodes for p-type SWCNT TFTs and interconnects, followed by annealing on a hot plate in air at 200 °C for 30 min.

All patterns were formed by using a Fujifilm Dimatix DMP-2800 printer with a drop volume of 10 pL cartridge. All printing steps were performed under ambient conditions in a cleanroom. The drop spacing was chosen to be 30 μm for Ag electrode printing and 40 μm for SWCNT and acetone printing. The substrates were heated at 60 °C for Ag electrode printing, but were unheated for SWCNT and acetone printing. Further details on the preparation of the SWCNT ink is described in previous work^48^.

### Electrical Characterization of Devices

All measurements were conducted under ambient conditions. The characterization of discrete TFTs and inverters was performed using a HP 4155 C semiconductor parameter analyzer. The output signals of ROSCs and NAND gates were measured with a LeCroy WaveRunner 6030 oscilloscope. The input signals for NAND gates were generated using a Tektronix AWG 2005 arbitrary waveform generator. DC biases for ROSCs and NAND gates were supplied by the HP 4155C.

For characterization as a sensor, saturated acetone vapor in nitrogen was delivered to the channel of p-type SWCNT TFTs through a syringe using a Manostat Vera Varistaltic Pump Plus with a flow rate of 20 mL min^−1^. More details on the experimental setup for acetone vapor delivery are described in ref. 45.

## Additional Information

**How to cite this article:** Kim, B. *et al*. Inkjet printed circuits based on ambipolar and p-type carbon nanotube thin-film transistors. *Sci. Rep.* **6**, 39627; doi: 10.1038/srep39627 (2016).

**Publisher's note:** Springer Nature remains neutral with regard to jurisdictional claims in published maps and institutional affiliations.

## Supplementary Material

Supplementary Information

## Figures and Tables

**Table 1:** Device characteristic parameters of the ambipolar and p-type (before and under acetone vapor exposure) SWCNT TFTs.

		mobility (cm^2^ V^−1^ s^−1^)	log (*I*_*on*_/*I*_*off*_)	*V*_*th*_(V)	S.S. (V/dec)
p-TFT	before acetone exposure	5.9 ± 1.7	1.77 ± 0.21	0.84 ± 0.07	1.67 ± 0.29
	under acetone exposure	7.3 ± 1.8	2.36 ± 0.25	0.79 ± 0.22	0.99 ± 0.25
Ambipolar TFT (n-channel operation)		2.4 ± 0.4	2.27 ± 0.23	1.75 ± 0.13	0.91 ± 0.17

The parameters were extracted from 6 different devices for ambipolar and p-TFTs.
