# Supplementary material for: Inkjet printed circuits based on ambipolar and p-type carbon nanotube thin-film transistors
Source: Sci Rep. 2017 Feb 1;7:39627. doi: 10.1038/srep39627 (PMC5286420; doi:10.1038/srep39627)
Supplement: Supplementary Information [file srep39627-s1.pdf]

Supplementary Information

Inkjet printed circuits based on ambipolar and p-type carbon nanotube thin-film transistors

Bongjun Kim[1,†,*], *Michael L. Geier*[2], *Mark C. Hersam*[2], *and Ananth Dodabalapur*[1,*]

[1]Microelectronics Research Center, The University of Texas at Austin, Austin, Texas 78758, United States

[2]Department of Materials Science & Engineering and Department of Chemistry, Northwestern University, Evanston, Illinois 60208, United State

[†]Current address: Cambridge Graphene Centre, University of Cambridge, Cambridge CB3 0FA, United Kingdom

[*]Correspondence and requests for materials should be addressed to B.K. (email: bongjun.kim@utexas.edu) or A.D. (email: ananth.dodabalapur@engr.utexas.edu).

Power dissipation of the inverter

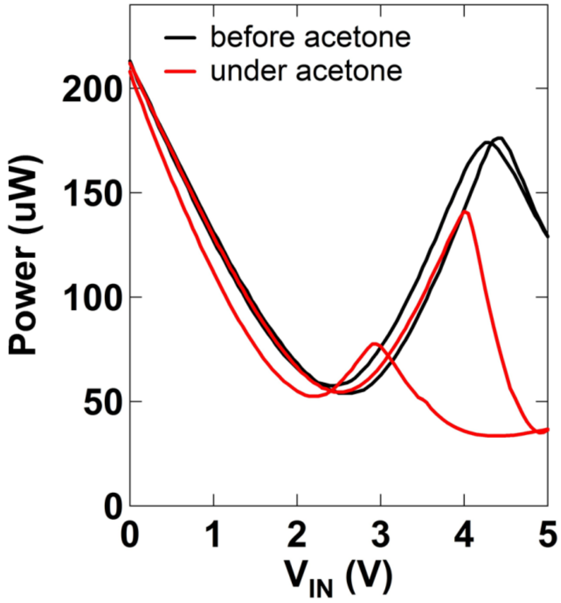

Figure S1. Power dissipation of the inverter based on ambipolar and p-type SWCNT TFTs before and under acetone vapor exposure.

Figure S1 shows the static power dissipation ($P = I_{DD}V_{DD}$) of the inverter based on ambipolar and p-type SWNCT TFTs before and under acetone vapor exposure. The power dissipation in complementary inverters typically shows a peak at a switching threshold ($V_{IN} = V_{OUT}$) and decreases at low and high V_{IN}, whereas the power dissipation in ambipolar inverters shows almost opposite trends (increases at low and high V_{IN}). The power dissipation in inverters based on ambipolar and p-type TFTs decreases at high V_{IN}, but increases at high V_{IN}.

Recovery characteristics of the inverter after acetone exposure

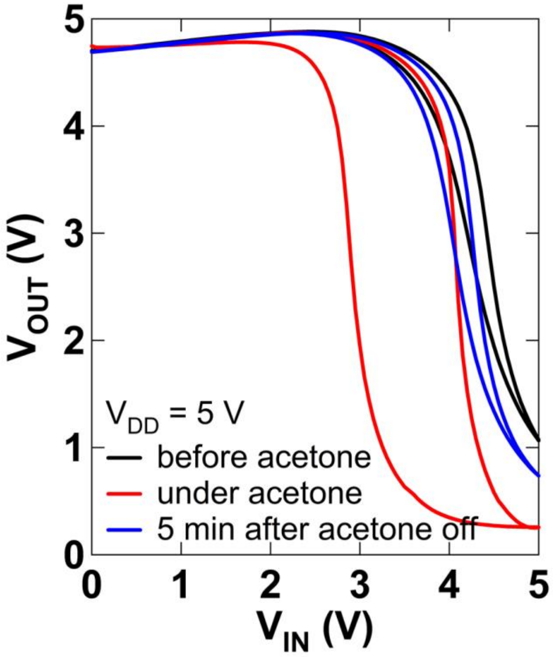

Figure S2. Voltage transfer characteristics of the inverter based on ambipolar and p-type SWCNT TFTs before and after acetone vapor exposure.

Figure S2 shows that the device characteristic changes in the inverter based on ambipolar and p-type SWCNT TFTs under acetone vapor exposure are reversible. The initial inverter characteristics before acetone vapor exposure were almost recovered 5 min after acetone flow was turned off under ambient conditions.
